# The trophic status of Suwałki Landscape Park lakes based on selected parameters (NE Poland)

**DOI:** 10.1007/s10661-014-3763-0

**Published:** 2014-05-01

**Authors:** Elżbieta Jekatierynczuk-Rudczyk, Piotr Zieliński, Magdalena Grabowska, Jolanta Ejsmont-Karabin, Maciej Karpowicz, Adam Więcko

**Affiliations:** Department of Hydrobiology, Institute of Biology, University of Białystok, Świerkowa 20B, 15-950 Białystok, Poland

**Keywords:** Trophic state index, Nutrients, Bacterioplankton, Phytoplankton, Zooplankton, Primary production

## Abstract

This study describes changes in the trophic status of 12 lakes within Suwałki Landscape Park (SLP). All of the trophic classifications of the lakes were based on the trophic continuum division. Trophic status was determined by means of multiparameter indices using several diverse criteria. In this study, the assessment of the trophic status of lakes included water quality; abundance and biomass of bacterioplankton, phytoplankton, and zooplankton; and primary production of phytoplankton. The Carlson trophic state index (TSI) describes the level of water fertility and indicated the dominance of moderately eutrophic waters. Lakes Perty, Jeglówek, and Hańcza have a trophic status that indicates mesotrophy (TSI <50). The trophic status of the studied lakes was determined based on the bacterial abundance and clearly showed a lack of oligotrophic lakes in SLP. Based on the number of bacteria, only Lake Szurpiły can be classified as β-mesotrophic, whereas Lake Linówek can be characterized as hypertrophic with some features typical for humic waters. The greatest value of gross primary production was observed in Lake Linówek (126.4 mg C/m^3^/h). The phytoplankton trophy index varied from 1.59 to 2.28, and its highest value, which indicated eutrophy, was determined for Lake Udziejek. In the case of Lakes Hańcza, Szurpiły, Perty, Jeglówek, and Kojle, the index ranged from 1.25 to 1.74, which indicated mesotrophy. The majority of the lakes were classified as mesoeutrophic (1.75–2.24). The highest trophic status was assessed for lakes with a marked dominance of cyanobacteria (Lake Przechodnie, Lake Krajwelek, Lake Udziejek, and Lake Pogorzałek), which is commonly recognized as an indicator of high trophic status. Considering all of the indices of trophic status, the analysis of rotifer community structure indicates that the studied group of lakes is mesoeutrophic or eutrophic. The values of crustacean zooplankton indices indicated that the trophic status of the studied lakes was close to that determined using a TSI. The parameters of zooplankton abundance and species structure allowed for the observance of changes in the tropic levels of lakes, which are difficult to detect by a chemical assay alone.

## Introduction

The Water Framework Directive 2000/60/EC commits the European Union member states to assess the ecological state of their surface and groundwaters; this assessment is based on biological elements, whereas hydromorphological, chemical, and physical features are treated as parameters that support the ecological state assessment (European Commission 2000).

Lakes are an important element of the natural environment that define both the landscape and its ecological functioning. In geological time, lake basins are short-lived and the eutrophication process is one of the reasons for their disappearance (Wetzel 2001). Lake eutrophication is the nutrient enrichment of lakes; every lake fits into a particular trophic status according to its degree of eutrophication, and all lakes change their trophic status over time. Lake eutrophication is caused by natural and anthropogenic human factors. Natural eutrophication is the process by which lakes gradually become aged and more productive, and it normally takes thousands of years to progress. However, most of these processes are accelerated by an excess of nutrients arising from human activity, which is referred to as “cultural eutrophication.” Cultural eutrophication is caused by an excess of plant nutrients (primarily N and P) disposed into the lakes mainly as untreated or partially treated domestic sewage, runoff from agricultural fields, etc. Phosphorus is the key nutrient for controlling eutrophication (Reynolds and Petersen 2000).

The trophic classification of lakes is based on the division of the trophic continuum. Trophic status is determined by multiparameter indices using several diverse criteria, such as the shape of the oxygen curve, species composition of bottom fauna or flora, concentration of nutrients, and measure of biomass (Michalski and Conroy 1972). In our study, the assessment of the trophic status of lakes included water quality; abundance and biomass of bacterioplankton, phytoplankton, and zooplankton; and primary production of phytoplankton.

The trophic status of lakes describes the development and functioning of aquatic organisms. Previous studies of the water chemistry and plankton structure of Lake Hańcza were conducted by Spodniewska (1978), Karabin and Ejsmont-Karabin (1993), Hillbricht-Ilkowska (1994), Hillbricht-Ilkowska and Wiśniewski (1994), Hutorowicz and Napiórkowska-Krzebietke (2008), Grabowska et al. (2006), and Zieliński et al. (2006). Other lakes within Suwałki Landscape Park, most of which are mesotrophic, have rarely been studied (Zieliński et al. 2006; Górniak et al. 2007). The total phosphorus load from the catchment of Suwałki Landscape Park (SLP) lakes is usually below the dangerous level (Hillbricht-Ilkowska 1994), which is why the eutrophication of these lakes is relatively slow.

Lakes in SLP are described as ecosystems with a high resistance to degradation (Bajkiewicz-Grabowska 1999). The nutrient load from the catchment was estimated as smaller than acceptable. Additionally, the catchment phosphorus load is characterized by the low availability of pelagic producers (Hillbricht-Ilkowska 1994). The uniqueness of these functional relations is a sufficient reason for them to be monitored. The aim of the study is to assess the trophic status of 12 lakes according to different biotic and abiotic parameters.

## Study area

**Fig. 1:**
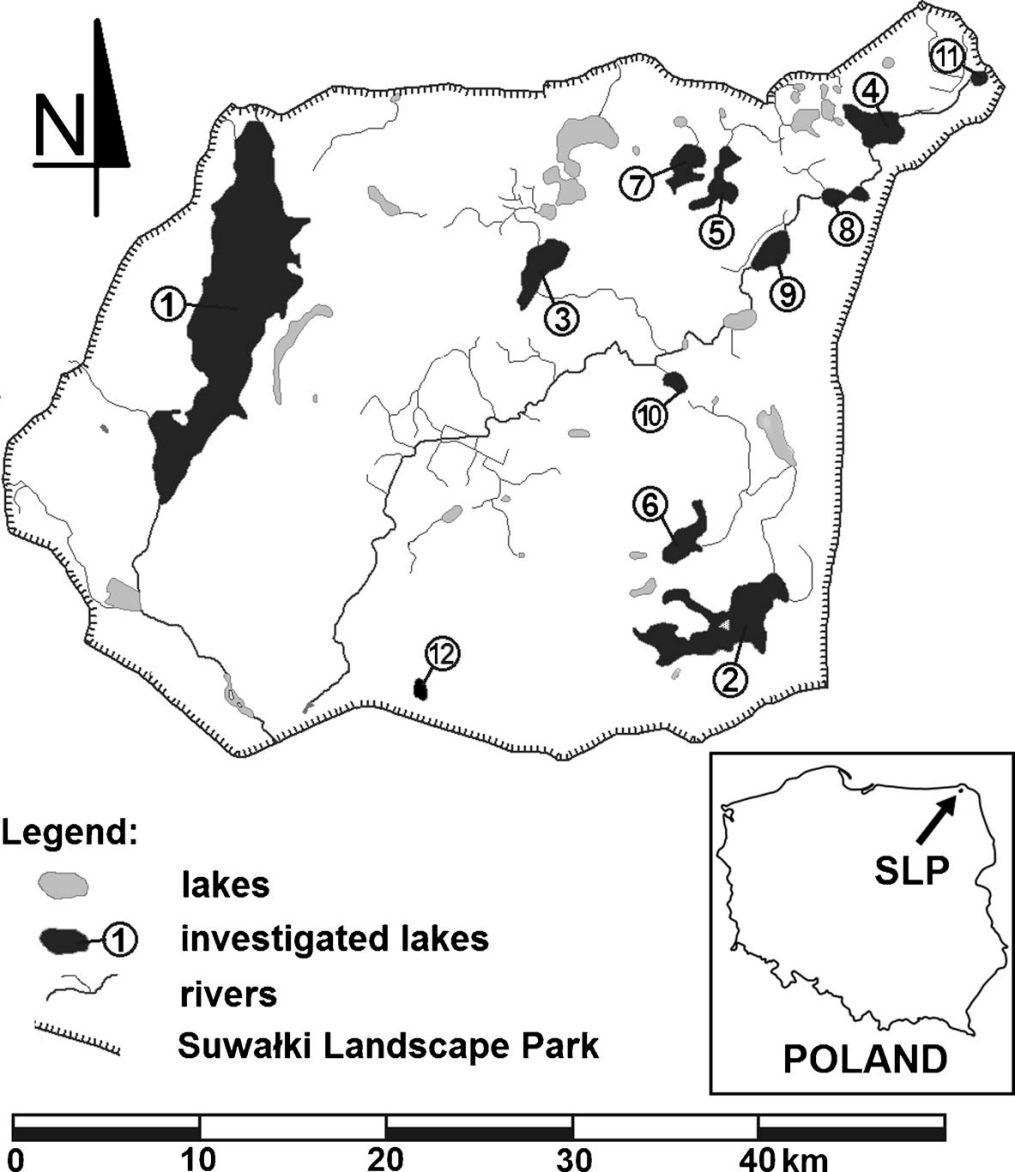
Localization of studied lakes in Suwałki Landscape Park. *1* Lake Hańcza, *2* Lake Szurpiły, *3* Lake Kamenduł, *4* Lake Przechodnie, *5* Lake Perty, *6* Lake Jeglówek, *7* Lake Kojle, *8* Lake Krajwelek, *9* Lake Okrągłe, *10* Lake Udziejek, *11* Lake Postawelek, *12* Lake Linówek

Suwałki Landscape Park was created in 1976 as the first landscape park in Poland. The park is located in the northeastern region of Poland in the province of Podlasie (Podlaskie Voivodeship). The area under investigation represents a typical landscape formed during the last Vistulian glacial. Nearly 10 % of SLP is covered with postglacial lakes (Ber 2000). This study included 12 SLP lakes located in the Lithuanian Lake District in the Niemen River catchment: Lake Hańcza, Lake Szurpiły, Lake Kamenduł, Lake Przechodnie, Lake Perty, Lake Jeglówek, Lake Kojle, Lake Krajwelek, Lake Okrągłe, Lake Udziejek, Lake Pogorzałek, and Lake Linówek (Fig. 1). The catchments of the lakes are mostly non-forested with extensive agriculture, and there is a population density of less than 30 persons per square kilometer. During summer, the SLP lakes are used for recreation. In the Suwałki District, 222 agritourist farms are registered, and they can significantly increase the population in summer, especially in the vicinity of the lakes.

**Fig. 2:**
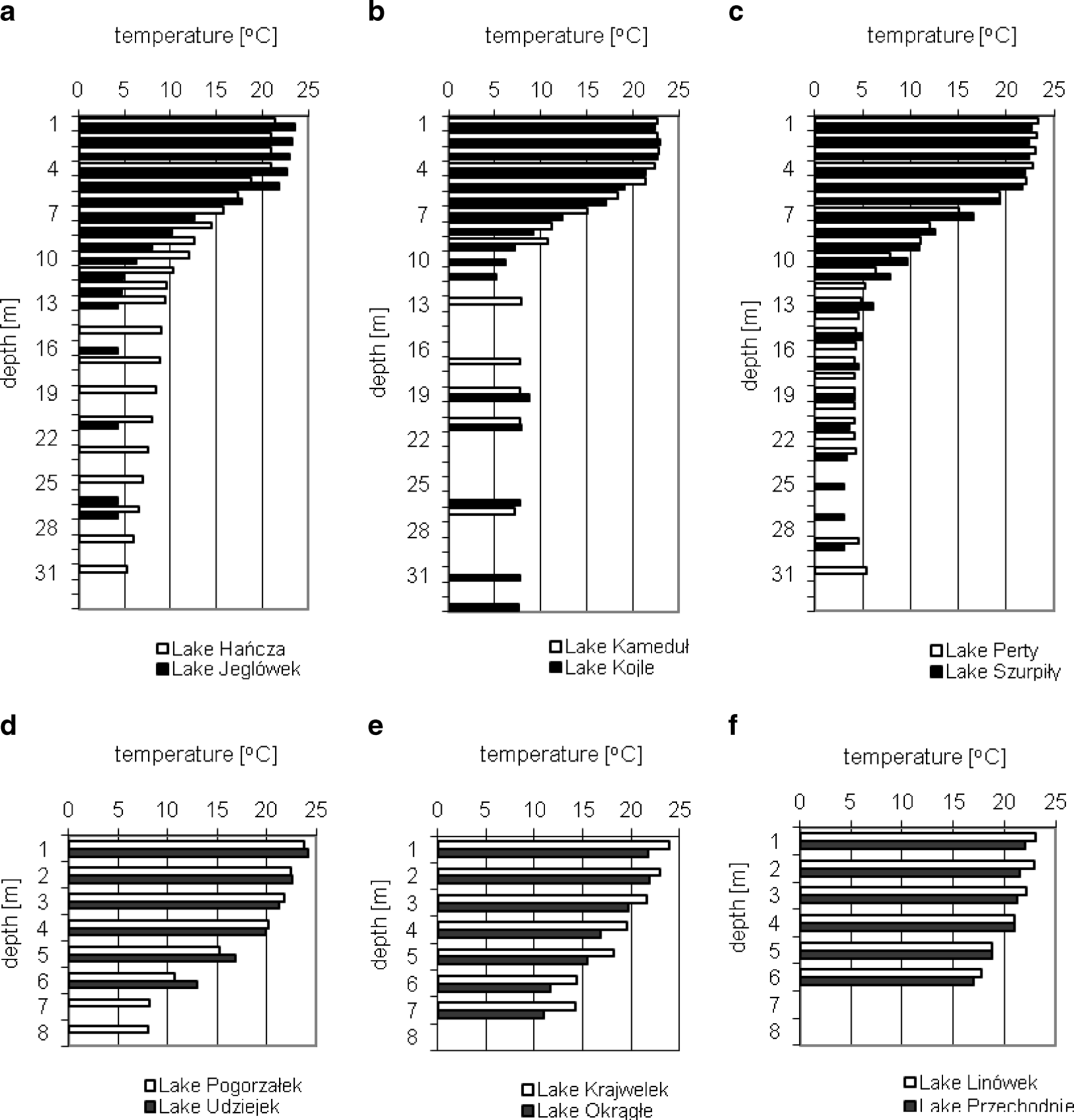
Vertical changes of water temperature in the lakes of Suwałki Landscape Park

The study was conducted in Lake Hańcza (the deepest lake in Poland); Lake Szurpiły (the maximum depth of which exceeds 45 m); Lakes Perty, Kojle, Kamenduł, and Jeglówek (the maximum depths of which exceed 15 m); and six shallow lakes with maximum depths not exceeding 10 m (Table 1). Eleven of these lakes have outflows and inflows, whereas Lake Linówek, which is located in the Szeszupa catchment, has no outflow. Lake Hańcza is located in the Czarna Hańcza catchment, and the remaining lakes are located in the Szeszupa catchment. Six of the lakes under study are thermally stratified (Lake Hańcza, Lake Jeglówek, Lake Perty, Lake Szurpiły, Lake Kamenduł, and Lake Kojle) (Fig. 2a–c), and the six shallow lakes are polymictic (Fig. 2d–f).

**Table 1:** Morphometric characteristics of the Suwałki Landscape Park lakes

Lakes	Latitude	Longitude	Surface of the lake [ha]	Volume of the lake [tys m^3^]	Maximum depth [m]	Average depth [m]	Length of coastline [m]	Shoreline development	Rate unveiling lake
Stratified lakes									
Hańcza	54° 15.9′	22° 48.9′	311.4	120,364.1	108.5	38.7	11,750	1.88	8.1
Szurpiły	54° 13.8′	22° 53.5′	80.9	8,168.0	46.0	10.0	7,000	2.19	8.1
Kamenduł	54° 16.0′	22° 52.0′	25.5	1,730.7	24.5	6.8	2,300	1.28	3.8
Perty	54° 16.5′	22° 53.9′	20.4	1,019.1	31.0	5.0	2,750	1.72	4.1
Jeglówek	54° 14.3′	22° 53.2′	19.2	1,444.0	18.1	7.5	2,585	1.66	2.6
Kojle	54° 16.8′	22° 53.3′	16.1	1,754.9	27.5	10.9	1,900	1.34	1.5
Polymictic lakes									
Przechodnie	54° 16.8′	22° 55.6′	25.4	848.6	6.3	3.3	2,150	1.20	7.7
Okrągłe	54° 16.1′	22° 54.4′	15.7	715.5	8.0	4.6	1,500	1.07	3.4
Krajwelek	54° 16.2′	22° 55.9′	15.8	474.0	6.0	3.3	n.d.	n.d.	n.d.
Udziejek	54° 15.1′	22° 53.2′	6.1	229.0	7.2	3.8	996	1.14	n.d.
Pogorzałek	54° 16.3′	22° 50.2′	3.5	92.0	5.4	2.6	n.d.	1.08	n.d.
Linówek	54° 13.5′	22° 50.3′	2.6^a^	70.0	5.6	2.7	625	n.d.	n.d.

*n.d.* no data

^a^Without heat

The complicated geological structure of the Suwałki Lake District is reflected in the geological types of its lakes and in the complex conditions that shape their hydrological and hydrogeological regimes. The studied lakes are diverse and include typical deep channel lakes such as Lake Hańcza, which has a maximum depth of 108.5 m and is the deepest lake in Poland and the Central European Lowlands; moraine lakes such as Lake Szurpiły, Lake Kojle, and Lake Perty, which have varied coastlines formed by the melting of dead ice blocks; and shallow heavily overgrown tanks-lakes such as Lake Linówek, which is gradually turning into a bog.

## Materials and methods

Summer water samples were taken on 23–31 July 2009 from the deepest parts of each lake. Samples for chemical, bacterioplankton, and phytoplankton analyses were collected at depths of 0.5, 7.0, and 30 m in Lake Hańcza; at 0.5, 7.0, and 22 m in Lake Szurpiły; at 0.5, 6.0, and 26 m in Lake Kamenduł; at 0.5, 6.0, and 20 m in Lakes Perty and Jeglówek; at 0.5, 6.0, and 32 m in Lake Kojle; at 0.5 and 4.0 m in Lake Przechodnie; at 0.5 and 6.0 m in Lakes Okrągłe and Krajwelek; at 0.5 and 5 m in Lake Udziejek; at 0.5, 4, and 7 m in Lake Pogorzałek; and at 0.5 and 4.5 m in Lake Linówek. Zooplankton was sampled with a 2-l sampler at every 1-m depth and pooled for the layers of epilimnion, metalimnion, and hypolimnion in stratified lakes and the epilimnion and a layer below it in shallow lakes. The samples were concentrated using a plankton net with a 30-μm mesh size and fixed in 4 % formalin. The field measurements included the Secchi disc visibility, water temperature, pH, oxygen saturation, and concentration of dissolved oxygen (HACH LANGE Sonde).

Laboratory analyses were conducted according to the methods described by Hermanowicz et al. (1999). The concentration of total phosphorus was determined after acidification and mineralization with UV radiation. The total nitrogen content was determined using a Tecator 2300 Kjeldahl analyzer. The dissolved organic carbon (DOC) content was determined using a Shimadzu total carbon analyzer (TOC) 5050A according to the procedure proposed by Zieliński and Górniak (1999). Chlorophyll *a* concentration was determined by the spectrophotometric method with ethanol extraction. Samples were filtered on GF/C filters and extracted with boiling 90 % ethanol (Lorenzen 1965; Nusch 1980).

To assess the trophic status of lakes on the basis of hydrochemical data, the Carlson index (Carlson 1977)—trophic state index (TSI) (TSI_SD_, TSI_Chl_, TSI_TP_)—and TSI_TN_ were used (Kratzer and Berezonik 1981).

The general number of bacteria cells (BN) was determined with epifluorescence microscopy. Formalin-preserved (2 % final concentration) and triplicate 4,6-diminido-2-phenylindole (DAPI)-stained samples were counted for bacterial abundance (Porter and Feig 1980). The biomass of bacteria (BB) was calculated for the number of bacteria according to the method by Theil-Nielsen and Sondergaad (1998). The bacterial trophic status was calculated according to Nixdorf and Jander (2003). In the method, bacterial cell number does not exceed 0.5 × 10^6^ cells/ml in oligotrophic lakes, 0.5–2 × 10^6^ cells/ml in mesotrophic lakes, 2–4 × 10^6^ cells/ml in eutrophic lakes, and 4 × 10^6^ cells/ml in hypertrophic ones.

Water samples (500–1,500 ml) for phytoplankton studies were immediately fixed using Utermöhl’s solution. Quantitative analyses were conducted in Fuchs-Rosenthal chambers, with counts including up to 200 individuals. The relationship between the size and the volume of phytoplankton cells was used to determine the biomass of algae.

The phytoplankton trophy index was based on the biomass of indicator taxa from the epilimnion of the lakes according to the equation of Hörnström (1981). Trophy index values (TI_PB_) for particular taxa were adopted following Szeląg-Wasilewska et al. (1999) and Szeląg-Wasilewska (2007). The phytoplankton trophy index values were assigned to six trophic states (0.00–0.74 oligotrophy, 0.75–1.24 oligomesotrophy, 1.25–1.74 mesotrophy, 1.75–2.24 mesoeutrophy, 2.25–2.74 eutrophy, 2.75–3.00 strong eutrophy).

Biomass of rotifers was established following Ejsmont-Karabin (1998). Six rotifer indices of lake trophy (Ejsmont-Karabin 2012) were used to establish the trophic state of the studied lakes. These were as follows: (1) rotifer numbers, (2) rotifer biomass, (3) percentage of bacterivorous species in total numbers of the community, (4) average individual body weight in the rotifer community, (5) percentage of the “tecta” form in the numbers of *Keratella cochlearis*, and (6) contribution of species indicative of high trophy in the numbers of all indicative species. It was assumed that the lakes with a TSI_ROT_ under 45 are mesotrophic, those with TSI_ROT_ values of 45–55 are mesoeutrophic, those with values 55–65 are eutrophic, and those with TSI_ROT_ above 65 are hypertrophic. Biomass of crustacean zooplankton was established following Bottrell et al. 1976. Three crustacean indices (TSI_CRU_) of lake trophy (Karabin 1985) were used to establish the trophic state of the studied lakes. These were as follows: TSI_CCB_—percentage of Cyclopoida in the total Crustacea zooplankton biomass, TSI_BR_—the ratio of biomass Cyclopoida to Cladocera, TSI_IS_—percentage of species indicative of high trophy in the indicative group’s numbers. It was assumed that the lakes with TSI_ROT_ and TSI_CRU_ under 45 are mesotrophic, those with TSI_ROT_ and TSI_CRU_ values of 45–55 are mesoeutrophic, those with 55–65 are eutrophic, and those with TSI_ROT_ and TSI_CRU_ above 65 are hypertrophic.

The primary production was assessed in Lake Hańcza, Lake Okrągłe, and Lake Linówek. Phytoplankton primary production measurements were carried out using oxygen method (Winberg 1960; Vollenweider 1969).

## Results and discussion

As a member state of the European Union, Poland has been obliged to implement the Water Framework Directive (European Commission 2000). One of the main goals of this directive is to achieve good water quality by 2015. The ecological and chemical states of waters should be assessed based on monitoring measurements.

**Fig. 3:**
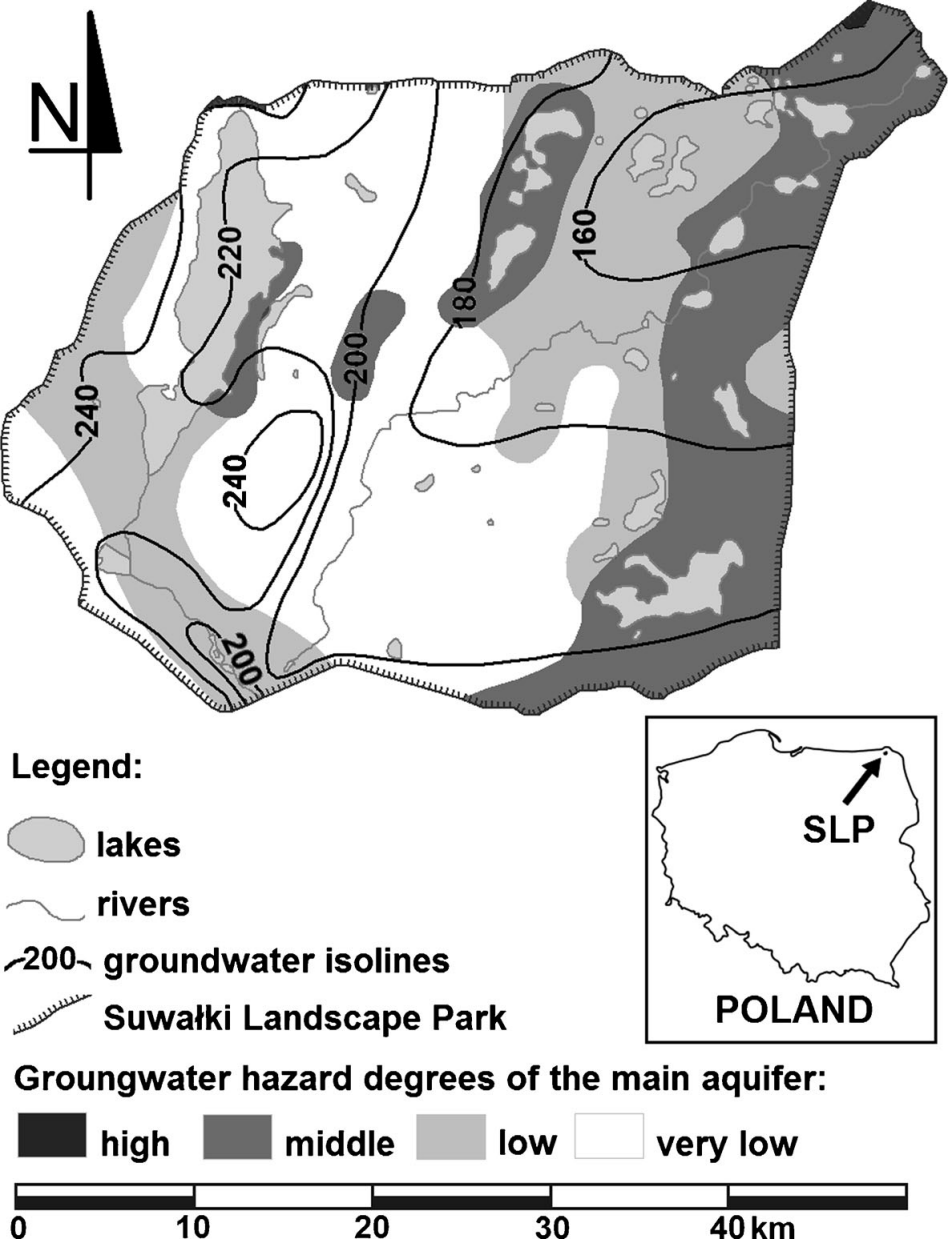
The hydroizohypse map of the first aquifer in Suwałki Landscape Park including areas susceptible to degradation

When assessing changes in the trophic status of the studied lakes, lakes with groundwater outflows cannot be ignored because the average groundwater outflow was estimated to be 4.5 l/s/km^2^ in the SLP area, and the quality of the groundwater can affect the trophic status of lakes. Based on the analysis of the geological and hydrogeological conditions, the degree of isolation of the main aquifer, contaminant migration time, type of land use, and location of potential pollution sources indicate different degrees of groundwater quality. Based on the analysis of the groundwater threat and hydraulic gradient of the aquifer, several areas of high susceptibility to trophic transformation can be distinguished in the Szeszupa catchment. An area with a moderate risk to the groundwater is on the eastern shore of Lake Hańcza; however, because of the decrease of underground water in an easterly direction, the groundwater does not adversely affect the lake trophy. The level of the absolute water surface in Lake Hańcza produces conditions for the infiltration of lake water into the groundwater area (Fig. 3).

The groundwater in the first aquifer is diionic (HCO_3_-Ca), with a composition characteristic of a shallow zone of intensive exchanges in the quaternary. The concentration of ammonium ions (N-NH_4_) (lower quartile–upper quartile 255–392 μg/l), nitrite (N-NO_2_) (lower quartile–upper quartile 3–16 μg/l), nitrate (N-NO_3_) (lower quartile–upper quartile 1,942–4,934 μg/l), soluble reactive phosphorus (SRP) (lower quartile–upper quartile 39–135 μg/l), and total phosphorus (TP) (lower quartile–upper quartile 437–616 μg/l) in the groundwater classifies it as the second and third class according to Polish norms; these classes indicate concentrations that are greater than in surface waters. The main sources of water pollution in the study area are discharged wastewaters, agriculture, and uncontrolled development of tourism (mainly agritourism).

In July 2009, the average water temperature in the SLP lakes was 15.6 °C, and it oscillated over a broad range (3.6–24.2 °C). The highest recorded temperature was in the surface layer of Lake Udziejek at 24.2 °C, whereas the lowest temperature was in the hypolimnion of Lake Szurpiły at 3.6 °C. Vertical changes in the water temperature varied (Fig. 2a–f). The decrease in temperature with depth in Lake Hańcza was lower than in the remaining stratified lakes, which can be associated with a large volume of water in the lake and slow heat loss (Table 2).

**Table 2:** The vertical variation of physicochemical parameters in the lakes of Suwałki Landscape Park in July 2009 (mean values ± standard deviation)

Nutrients		Stratified lakes			Polymictic lakes	
		Epilimnion	Metalimnion	Hypolimnion	Surface layer	Near-bottom layer
*T*	°C	22.48 ± 0.89	12.95 ± 1.52	5.33 ± 1.67	22.07 ± 3.15	13.97 ± 4.19
pH		8.28 ± 0.34	7.83 ± 1.17	7.63 ± 0.26	8.24 ± 0.43	7.59 ± 0.30
Eh	mV	229 ± 58	229 ± 58	232 ± 59	213 ± 72	219 ± 59
EC	μS/cm	319 ± 46	354 ± 59	359 ± 59	305 ± 128	365 ± 138
Oksygen	mg/l	9.36 ± 0.39	8.75 ± 3.27	2.62 ± 2.88	8.64 ± 3.12	2.25 ± 2.67
Saturation	%	109 ± 3	89 ± 32	21 ± 24	102 ± 42	25 ± 32
Chl a	μg/l	1.44 ± 0.52	3.27 ± 2.78	1.59 ± 1.79	6.02 ± 2.71	20.17 ± 8.63
TOC	mg/l	14.59 ± 6.52	13.93 ± 5.54	12.95 ± 5.84	19.28 ± 3.46	19.16 ± 4.21
DOC	mg/l	12.3 ± 5.15	11.85 ± 4.27	10.73 ± 3.32	16.92 ± 2.82	15.93 ± 3.39
POC	mg/l	2.27 ± 1.86	2.09 ± 2.44	2.22 ± 2.65	2.35 ± 1.69	3.23 ± 3.11
N-NH_4_^+^	μg N/l	175 ± 81	183 ± 129	442 ± 297	200 ± 147	313 ± 272
N-NO_2_^−^	μg N/l	4 ± 2	6 ± 5	4 ± 2	4 ± 4	6 ± 5
N-NO_3_^−^	μg N/l	56 ± 24	51 ± 13	116 ± 94	51 ± 14	58 ± 20
TON	μg N/l	443 ± 395	688 ± 457	340 ± 265	370 ± 138	830 ± 536
TN	μg N/l	678 ± 372	927 ± 366	786 ± 388	624 ± 177	1,206 ± 710
DP	μg P/l	73 ± 42	86 ± 59	81 ± 39	96 ± 48	112 ± 56
SRP	μg P/l	29 ± 32	34 ± 32	22 ± 15	29 ± 15	34 ± 12
ORP	μg P/l	44 ± 28	52 ± 62	58 ± 36	68 ± 44	78 ± 48
PP	μg P/l	52 ± 47	24 ± 15	69 ± 29	27 ± 28	37 ± 20
TP	μg P/l	125 ± 85	110 ± 64	150 ± 64	123 ± 56	149 ± 66
N/P		11	14	6	6	9

In the surface waters of the studied lakes, the electrolytic conductivity (EC) ranged from 166 to 427 μS/cm. The water conductivity increased with depth from the hypolimnion to near-bottom layer and reached an average value of 361 μS/cm. The hypolimnion and near-bottom waters showed a pH (7.57 and 7.68, respectively) that was almost 1 unit lower than that of the surface layers of lakes. In all of the lakes, the water pH value exceeded 7.00. The lowest value of redox potential (Eh) was observed in the epilimnion of Lake Hańcza (117.7 mV), and the largest value exceeded 300 mV in Lake Krajwelek. The water in the SLP lakes was diionic (HCO_3_-Ca).

**Fig. 4:**
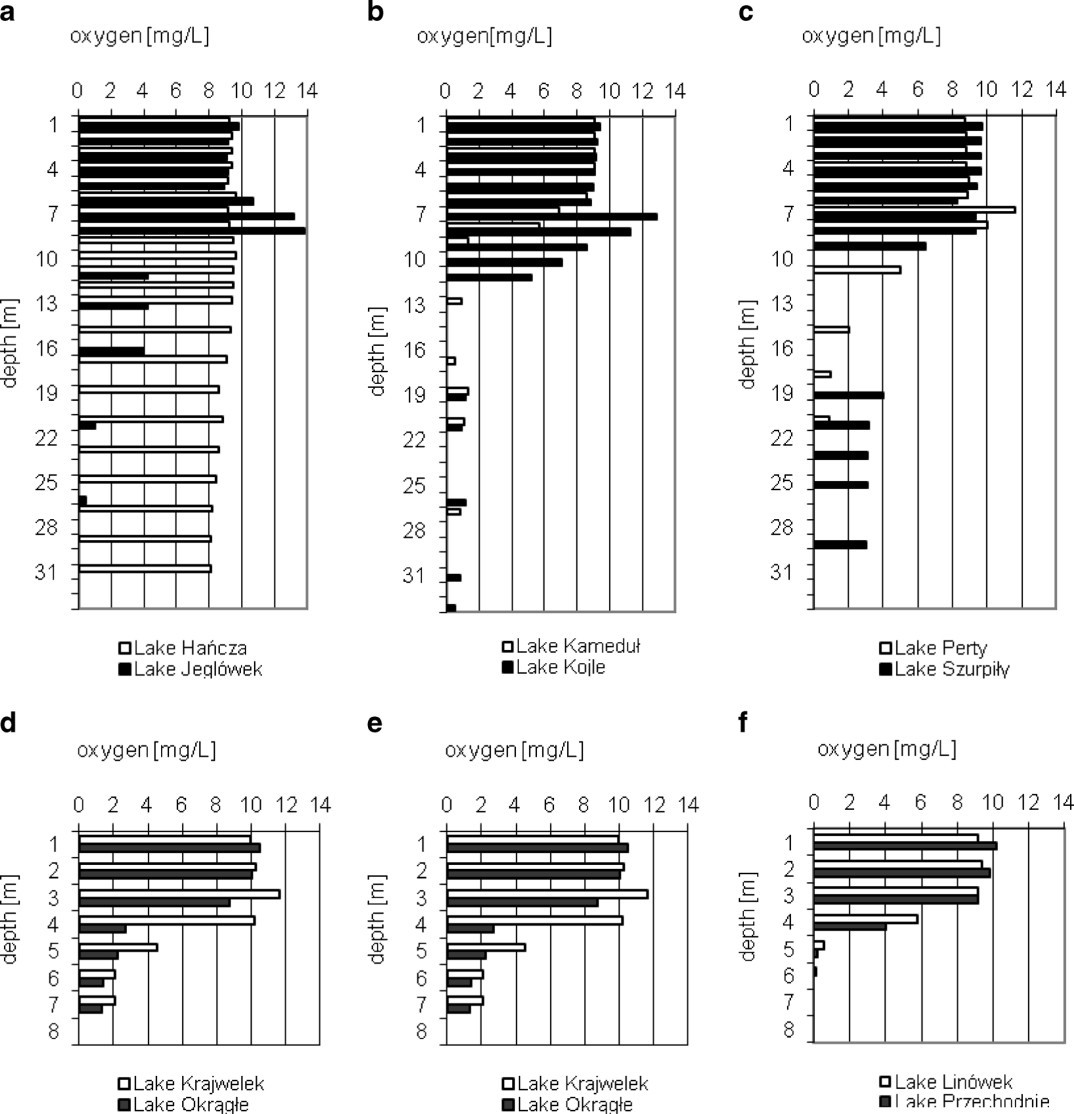
Vertical changes of the oxygen concentration in pelagial waters in the lakes of Suwałki Landscape Park

In four lakes, oxygen was detected in bottom layers (Fig. 4a–f). Oxygen saturation of the hypolimnion waters in Lake Hańcza exceeded 65 %. In Lakes Jeglówek, Kojle, Perty, and Krajwelek, the vertical distribution of oxygen was typical of a positive heterograde oxygen curve, whereas in the remaining lakes, it was typical of a klinograde oxygen curve (Fig. 4a–f). The average concentration of total phosphorus in the lake water was 130 mg/l, which was much lower than in previous periods (Table 2) (Górniak et al. 2007).

In the water of the SLP lakes, ammonium nitrogen was the dominant form of mineral nitrogen. The maximum value of N-NH_4_ (841 μg/l) was recorded in the hypolimnion of Lake Kamenduł. The concentration of ammonia nitrogen increased with depth (Table 2), which was similar to the changes in oxygen concentration. The correlation between the concentration of oxygen and N-NH_4_ was −0.37 (*p* < 0.05). In the hypolimnion of Lake Kojle, the ammonia nitrogen concentration also exceeded 800 μg/l, whereas the oxygen saturation did not exceed 5 %. The nitrite nitrogen concentration in Lake Linówek exceeded 10 μg/l, and much of the immediate catchment area of this lake is used as pasture. In most of the other lakes, the N-NO_2_ concentration oscillated between 0 and 5 μg/l. In the surface layer of the lakes, a low concentration of nitrate nitrogen compared to ammonia nitrogen was found. The average concentration of N-NO_3_ in the surface layer of the SLP lakes did not exceed 55 μg/l. The average concentration of nitrate nitrogen in the hypolimnion of the analyzed lakes exceeded 110 μg/l. The highest concentration of nitrate was found in the hypolimnion of Lake Hańcza (244 μg/l) and Lake Szurpiły (229 μg/l). In most of the SLP lakes, the concentration of nitrate nitrogen increased with depth; however, an increase with depth was recorded in two lakes: Kamenduł and Perty. In the metalimnion of the stratified lakes, the highest concentrations of total nitrogen (TN) (927 μg/l) and organic nitrogen (TON) (687 μg/l) were recorded. In the polymictic lakes, the total nitrogen content in the surface layer was 624 μg/l, whereas in the bottom layer, it exceeded 1,200 μg/l. The average concentrations of organic nitrogen in the surface and bottom layers were 370 and 829 μg/l, respectively. The concentration of TOC in the SLP lakes ranged from 7.4 to 24.6 mg/l, which accounted for over 85 % of the DOC and for 15 % of the suspended organic carbon (POC). The concentrations of organic carbon forms in the water column were uniform.

The average concentration of chlorophyll *a* in the water of the SLP lakes fluctuated around 6 μg/l. Low concentrations of chlorophyll in the epilimnion of Lake Szurpiły and Lake Hańcza were also shown by the data collected for the years 1963–2005 (Hillbricht-Ilkowska 1994; Grabowska et al. 2006).

Lakes in SLP represent diverse chemical compositions and trophic statuses (Hillbricht-Ilkowska and Wiśniewski 1994; Górniak et al. 2007). The mean value of the index calculated according to the Carlson formula was 55. The average value of the TSI index based on the visibility of the Secchi discs, concentration of chlorophyll *a*, and total nitrogen was approximately 45. The average value of TSI_TP_ (74) was markedly higher in all of the studied lakes (Table 3) and ranged from 58 in Lake Perty to 82 in Lake Udziejek. The spatial variability of TSI_TP_ was the smallest, with a coefficient of variation (CV) = 9 %. The greatest spatial variability was characterized by TSI_Chl_ (CV = 29 %). The TSI_SD_ values also indicated spatial variation (CV = 12 %). The average value of TSI_SD_ in the analyzed lakes was low (Table 3). In three lakes (Lake Hańcza, Lake Perty, and Lake Jeglówek), the TSI_SD_ value did not exceed 40. The largest value of this index (55) was calculated for Lake Przechodnie, which is situated in the peat valley Szeszupa and loaded with high amounts of organic matter (DOC = 17.5 mg/l).

**Table 3:** Trophic indices in the lakes of Suwałki Landscape Park (1983/1985—Hillbricht-Ilkowska and Wiśniewski 1994; 2003—Górniak et al. 2007; 2009—own research)

Lakes	TSI_SD_			TSI_Chl_			TSI_TP_			TSI			TSI_TN_	N/P
	1983/1985	2003	2009	1983/1985	2003	2009	1983/1985	2003	2009	1983/1985	2003	2009	2009	
Hańcza	29	34	38	8	21	35	55	71	69	31	42	47	54	12
Szurpiły	38	49	47	32	38	36	54	90	79	41	59	54	35	3
Kamenduł	46	47	48		36	42	69	78	77	58	54	56	55	7
Perty	39	42	38	27	21	23	44	66	58	37	43	40	42	8
Jeglówek	34	43	37	32	32	31	50	81	65	39	52	44	55	26
Kojle	36	47	45	41	30	38	54	78	80	44	52	54	41	12
Przechodnie	54	n.a.	55	62	n.a.	49	60	n.a.	75	59	n.a.	60	40	2
Okrągłe	52	n.a.	50	n.a.	n.a.	55	61	n.a.	77	57	n.a.	61	50	7
Krajwelek	53	59	53	68	45	55	63	80	78	61	61	62	53	10
Udziejek	51	58	52	66	56	55	60	78	82	59	64	63	47	3
Pogorzałek	n.a.	55	54	n.a.	43	69	n.a.	81	75	n.a.	60	66	45	7
Linówek	n.a.	45	45	n.a.	37	41	n.a.	79	68	n.a.	53	51	42	7

*n.a.* not analyzed

TSI_SD_ changes in recent years were lower (Table 3), and special attention was paid to the stability of some of the water characteristics in the SLP lakes. Stability of this characteristic applies to Lake Hańcza, Lake Jeglówek, Lake Szurpiły, Lake Kamenduł (Hillbricht-Ilkowska and Wiśniewski 1994). Research carried out at the beginning of the twenty-first century confirmed the previous observations and allowed including Lake Kojle to a group of lakes, which are characterized by constant water transparency (Table 3). Hillbricht-Ilkowska and Wiśniewski (1994), based on their own and literature research, found a decrease in water transparency in the lakes of the Szeszupa system. Our studies have shown the relative stability of this characteristic and that the trophic state index calculated based on Secchi’s disc visibility in Lake Krajwelek oscillated around 50. The trophic state index calculated for the concentration of chlorophyll *a* also showed spatial diversity. The average value of this index in the studied lakes was 44. Its lowest value (i.e., 23) was observed in Lake Perty. The River Szeszupa flows through Lakes Okrągłe, Krajwelek, Przechodnie, and Udziejek, and the TSI_Chl_ values of these lakes exceeded 55.

**Fig. 5:**
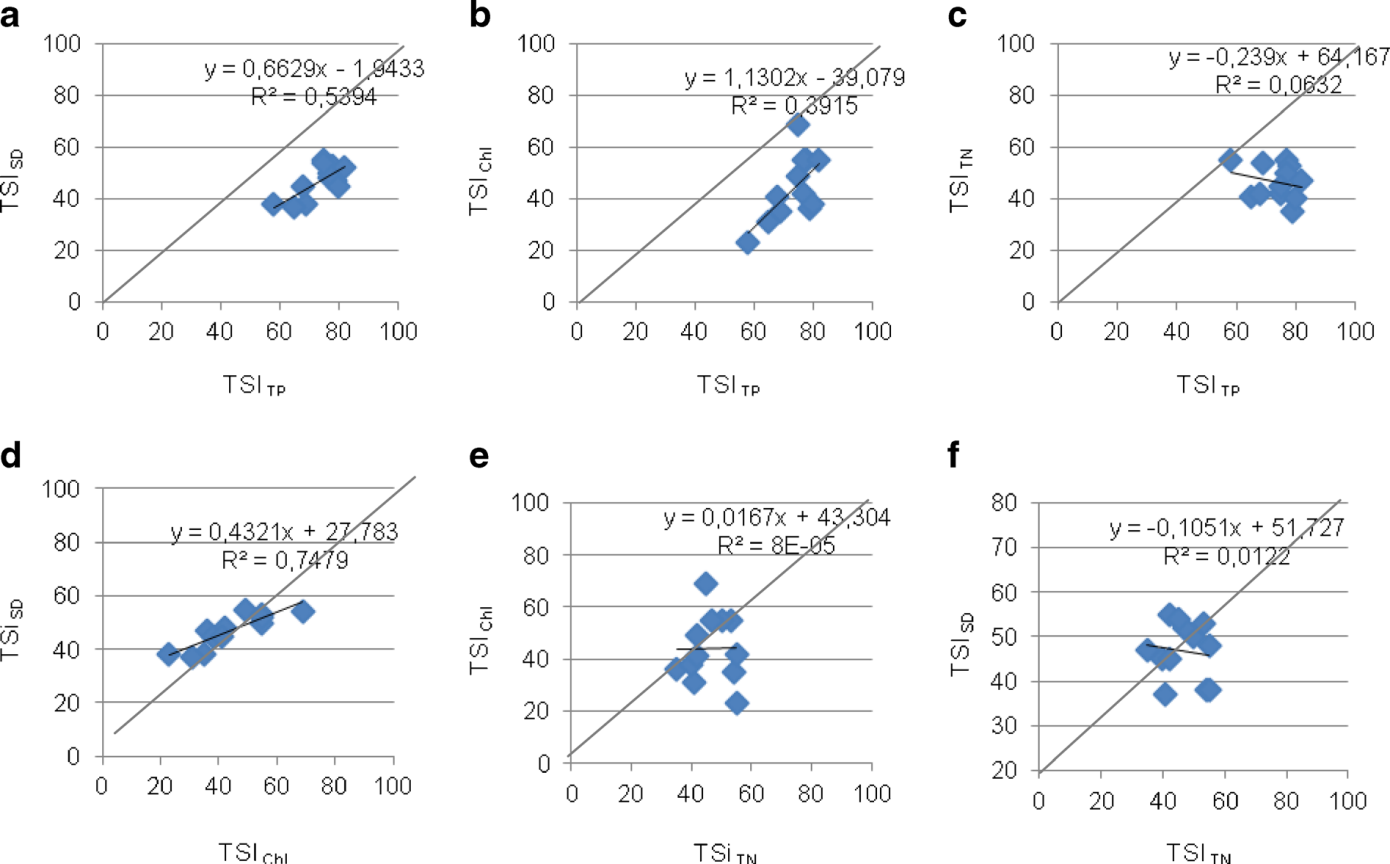
Comparison of the trophic state index values in the lakes of Suwałki Landscape Park: **a** TSI_TP_-TSI_SD_, **b** TSI_TP_-TSI_Chl_, **c** TSI_TP_-TSI_TN_, **d** TSI_Chl_-TSI_SD_, **e** TSI_TN_-TSI_Chl_, and **f** TSI_TN_-TSI_SD_

Carlson’s trophic state index was calculated for the SLP lakes and indicated that the moderately eutrophic waters were dominant except in the three mesotrophic (TSI <50) lakes, which were Lakes Perty, Jeglówek, and Hańcza. In accordance with the suggestions of Hillbricht-Ilkowska and Wiśniewski (1994) and Górniak et al. (2007), the calculated TSI values based on the total phosphorus concentration were significantly different from the values based on the chlorophyll *a* concentration and Secchi visibility. This result suggests that the majority of the limnic waters are currently mesotrophic or mesoeutrophic. In the studied lakes, a high TP concentration did not significantly affect the development of phytoplankton because the majority of the TP was in an unavailable form. The SRP in the lake water of the Szeszupa system often constituted less than 20 % of the TP. Such a situation was most likely related to the character of the soils in the catchments of lakes, which produce strong phosphorus complexes that make phosphorus unavailable for autotrophs (Hillbricht-Ilkowska 1994). The available phosphorus resources can also be restricted as a result of complexation by calcium, aluminum, and iron compounds, which can be subjected to sedimentation (Zdanowski 1983). The divergence from accepted principles for determining the trophic status of lakes based on the partial trophic index confirmed the relationship between them (Fig. 5a–f). In theory, all indicators should be equal, and the points might be grouped on or near the diagonal (Matthews et al. 2002; Bajkiewicz-Grabowska 2007; Jarosiewicz and Fryda 2011).

In July 2009, high water levels were recorded in the SLP lakes, which were a reaction to high rainfall in May and June 2009 (Jekatierynczuk-Rudczyk et al. 2012). The total precipitation in June 2009 was almost 150 % of the total precipitation in June from the years 1965–1995. The high summer rainfall caused a decrease in nutrient concentrations in the surface layers of the stratified lakes (Table 2) (Jekatierynczuk-Rudczyk et al. 2012).

**Fig. 6:**
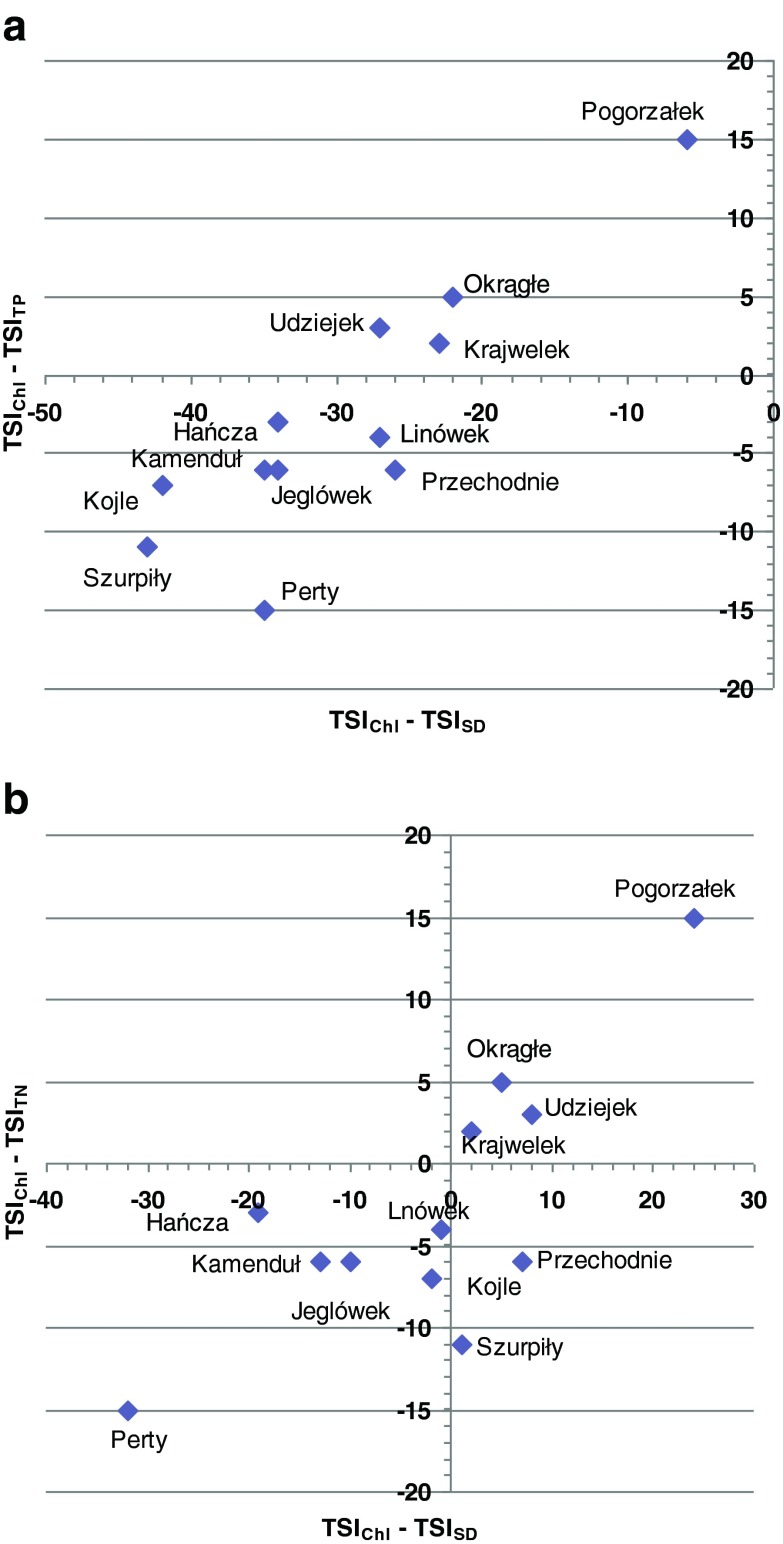
Diagram of the differences between trophic state indices in the lakes of the Suwałki Landscape Park. **a** relation TSIChl-TSISD and TSIChl-TSITP **b** relation TSIChl-TSISD and TSIChl-TSITN

Identification of a specific ecosystem is possible by calculating the differences between the trophic indices (Jarosiewicz and Fryda 2011). Therefore, it can be stated that in Lakes Pogorzałek, Okrągłe, Udziejek, and Krajwelek, productivity was limited by the availability of nutrients (Fig. 6a and b). The study by Hillbricht-Ilkowska and Wiśniewski (1994) showed that the weight ratio of nitrogen to phosphorus in the lakes of the SLP was close to 20, which suggests a relative deficit of phosphorus and may be the reason for low trophic status of the lakes. The calculated weight ratio of nitrogen to phosphorus in the surveyed lakes in July 2009 was much lower (average 8.6). The smallest value of N to P was found in Lake Przechodnie (2), and the highest value was found in Lake Jeglówek (26). A low N to P ratio suggests that nitrogen may be a limiting factor in phytoplankton growth. In northeastern Poland, such situations are observed in spring (Dunalska 2010; Napiórkowska-Krzebietke and Hutorowicz 2008).

The density of bacterial cells is a sensitive indicator of the processes that occur in all water bodies, which makes them a good determinant of the ecological status of lakes (Świątecki 1997; Górniak 2010). The average integrated bacterial number in the SLP lakes was 2.97 × 10^6^ cells/ml, and the bacterial biomass averaged 104.63 μG C/l. Their total numbers varied from 1.6 × 10^6^ cells/ml in deep Lake Kojle to 5.2 × 10^6^ cells/ml in dystrophic Lake Linówek (Table 4). Four types of vertical distribution of bacterioplankton were observed in the stratified lakes: decrease of bacterioplankton abundance with lake depth (Lake Hańcza and Lake Jeglówek), increase of bacterial abundance with lake depth (Lake Kojle and Lake Pogorzałek), maximum bacterial abundance in the metalimnion (Lake Perty), and minimum bacterial abundance in the metalimnion (Lake Szurpiły and Lake Kamenduł).

**Table 4:** Bacterial numbers (BN) and bacterial biomass (BB) in the lakes of Suwałki Landscape Park (July 2009)

Lakes	Depth [m]	BN [million cells/ml]	Average BN [million cells/ml]	Average BB [μg C/l]	BN (2003) Zieliński et al. (2006)
Hańcza	2	3.4	2.45	85.6	3.56
	7	2.1			
	30	1.9			
Szurpiły	0.5	1.9	1.65	65.7	3.7
	6	0.8			
	22	2.3			
Kameduł	0.5	3.3	3.37	96.5	5.02
	6	2.4			
	26	4.4			
Perty	0.5	2.1	2.38	83.3	4.24
	6	3.5			
	20	1.6			
Jeglówek	0.5	3.8	3.07	107.7	3.17
	6	3.0			
	20	2.5			
Kojle	0.5	2.4	3.30	115.4	2.18
	6	3.4			
	32	4.0			
Przechodnie	0.5	3.2	3.06	107.1	n.a.
	4	3.0			
Krajwelek	0.5	3.8	3.46	121.1	5.65
	6	3.1			
Okrągłe	0.5	2.2	3.34	116.9	n.a.
	6	4.5			
Udziejek	0.5	3.5	3.10	107.0	5.75
	5	2.7			
Pogorzałek	0.5	1.9	2.79	97.6	5.97
	4	2.8			
	7	3.7			
Linówek	0.5	5.2	4.80	168.0	4.94
	4.5	4.4			

*n.a.* not analyzed

The trophic status of the investigated lakes in Suwałki Landscape Park determined by the number of bacteria (Wetzel 2001; Nixdorf and Jander 2003) indicated a lack of oligotrophic lakes in the area (Table 4). However, the bacterial number of Lake Szurpiły classified it as β-mesotrophic, and Lake Linówek was characterized as hypertrophic with some features typical of humic waters (Table 4).

**Fig. 7:**
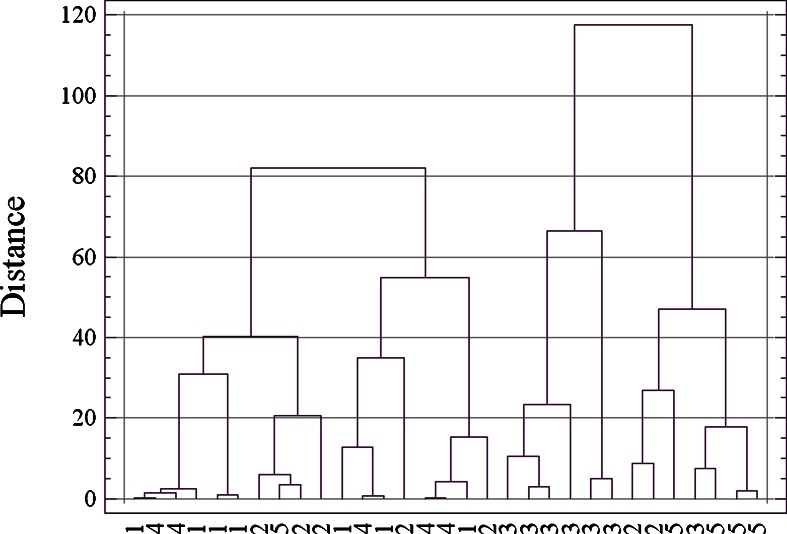
Variation of the bacterial numbers in the lakes of Suwałki Landscape Park (*1* epilimnion, *2* metalimnion, *3* hypolimnion, *4* surface layer, *5* bottom layer of water)

Additional explanations for the higher trophic level of Lake Linówek were the intensive grazing that occurred in the direct lake catchment area and the large slope rate of the pasture. The greatest number of bacterial cells was most frequently observed in the shallow lakes because their characteristics of higher temperatures, frequent mixing of the water column, and presence of other organisms create favorable conditions for the development of bacterioplankton (Fig. 7).

Kiliańczyk et al. (2002) found that lakes with higher temperatures and frequent mixing are characterized by elevated bacterial numbers originating from the benthic sediment layer. These features, as well as a large amount of biogenic elements such as nitrogen and phosphorus, make these lakes the richest in bacterial assemblages.

In two stratified lakes (Lake Kamenduł and Lake Kojle), the largest number of bacteria was found in the hypolimnion (Table 4), which may have resulted from the content of the disturbed bottom sediments that was taken in the water sample. Some researchers believe that bacteria affect the oligotrophication of lake waters and participate in the self-purification process of water. The deep lakes (Lake Hańcza, Lake Szurpiły, and Lake Perty) had the lowest density of bacterioplankton, which did not exceed 2.5 million cells/ml.

Our studies confirm the results presented by Zieliński et al. (2006), who found a lack of oligotrophic lakes in Suwałki Landscape Park in 2003. In 2009, a significantly lower number and biomass of bacterioplankton were found in the SLP lakes compared to the Masurian Lake District. Górniak (2010) found that in Lake Kuc (the Masurian Lake District), bacterial numbers can reach values of up to 11 million cells/ml. Another factor influencing the higher numbers of bacteria in the SLP lakes includes the heavy rainfall period in May and June 2009 (Jekatieryńczuk-Rudczyk et al. 2012).

In the 12 lakes of SLP, 158 phytoplankton taxa belonging to nine algal groups were found. Representatives of the four phytoplankton groups Cryptophyta, Bacillariophyceae, Chrysophyceae, and Chlorophyceae were found in all of the examined lakes. Four other groups, Cyanoprokaryota (Cyanobacteria), Dinophyta, Euglenophyta, and Zygnemathophyceae, were present in most of the studied lakes. Xanthophyceae was only present in Lake Hańcza, Lake Szurpiły, and Lake Przechodnie.

The total phytoplankton biomass (PB) fluctuated from 0.17 mg/l (Lake Linówek) to 8.15 mg/l (Lake Udziejek). Higher PB values were recorded in the stratified lakes compared to the polymictic shallow lakes. In the surface layer of four shallow lakes (Lake Przechodnie, Lake Krajwelek, Lake Udziejek, and Lake Pogorzałek), the PB exceeded 2 mg/l, and the share of filamentous cyanoprokaryotes in the PB exceeded 40 % of PB (Table 5). In the remaining shallow lakes, the domination of cryptophytes in Lake Linówek and the codomination of two algal groups in Lake Okrągłe were determined (Table 5), and similar results were recorded for the humic Lake Gorbacz (Zieliński et al. 2011). In the epilimnion of the stratified lakes, the main components of PB were diatoms (Lake Hańcza and Lake Kojle), chlorophytes (Lake Szurpiły), and chrysophytes (Lake Perty). Lakes codominated by chrysophytes and diatoms (Lake Jeglówek) or dinoflagellates and diatoms (Lake Kameduł) were recorded. Among the cyanoprokaryotes, the most frequent occurrences were of *Aphanizomenon flos-aquae*, *Aphanizomenon gracile*, *Cuspitothrix issatschenkoi*, *Dolichospermum*, *Anabaena flos-aquae*, and *Dolichospermum planctonicum*. Diatoms were mainly represented by centric forms from the genus *Cyclotella* (*Cyclotella meneghiniana*, *Cyclotella radiosa*, and *Cyclotella* sp.). Dinoflagellates were represented mainly by *Ceratium furcoides*, *Ceratium hirundinella*, *Peridiniopsis cunningtonii*, *Peridiniopsis elpatiewskyi*, and *Peridinium gatunense*, whereas chrysophytes were represented by *Dinobryon bavaricum*, *Dinobryon divergens*, *Dinobryon sertularia*, and *Dinobryon sociale*.

**Table 5:** Phytoplankton parameters in the studied lakes’ epilimnion of Suwałki Landscape Park in 2009 compared with summer total phytoplankton biomass in 10 lakes from the years 2003 (Grabowska et al. 2006)

Lakes	The most important taxa in phytoplankton biomass with theircontribution (%)	Biomass in this study [mg/l]	Biomass in 2003 [mg/l]	TI_PB_
Stratified lakes				
Hańcza	*Cyclotella radiosa* (55.8 %)	0.73	0.17	1.67
Szurpiły	*Phacotus lenticularis*, *Oocystis* spp. (41.2 %)	0.35	1.99	1.59
Kamenduł	*Ceratium hirundinella* (26.2 %), *Cyclotella* spp. (23.6 %)	0.64	1.33	2.01
Perty	*Dinobryon* spp. (43.2 %)	0.23	0.84	1.68
Jeglówek	*Dinobryon* spp. (29.4 %), *Cyclotella* spp. (19.6 %)	0.21	2.99	1.71
Kojle	*Cyclotella* spp. (53.3 %)	0.54	1.79	1.68
Polymictic lakes				
Przechodnie	*Aphanizomenon* spp. (46.0 %)	4.48	n.a.	2.15
Okrągłe	*Peridiniopsis* spp. (13.2 %), *Peridinium gatunense* (11.5 %), *Anabaena* spp. (12.4 %), *Cuspidothrix issatschenkoi* (12.4 %)	0.73	n.a.	2.03
Krajwelek	*Aphanizomenon* spp. (23.0 %), *Dolichospermum* spp. (19.1 %)	3.83	4.43	2.15
Udziejek	*Aphanizomenon* spp. (75.7 %)	8.15	n.d.	2.28
Pogorzałek	*Aphanizomenon* spp. (45.6 %)	2.53	2.33	2.11
Linówek	*Rhodomonas* sp., *Cryptomonas* spp. (42.9 %)	0.17	n.a.	2.09

*n.a.* not analyzed

Phytoplankton parameters are excellent indices of a water’s trophic status (Hörnström 1981; Hutchinson 1967; Szeląg-Wasilewska et al. 1999; Reynolds et al. 2002; Szeląg-Wasilewska 2007). The phytoplankton trophy index (TI_PB_) varied from 1.59 to 2.28, which are values typical of mesotrophy, mesoeutrophy, and eutrophy (Table 5). The highest index ascribed to eutrophy was determined for Lake Udziejek. In Lakes Hańcza, Szurpiły, Perty, Jeglówek, and Kojle, the trophic index was within the range of 1.25–1.74 and indicated that these lakes represented mesotrophic states (Table 5). The majority of lakes (6) were classified as mesoeutrophic (1.75–2.24). Species that form abundant populations had a large influence on the trophic index. The highest trophy occurred for lakes with a clear dominance of cyanobacteria (Lake Przechodnie, Lake Krajwelek, Lake Udziejek, and Lake Pogorzałek), which is a commonly recognized indicator of the high trophic status of water (Reynolds et al. 2002; Szeląg-Wasilewska et al. 1999; Szeląg-Wasilewska 2007). Additionally, the presence of *Trachelomonas* and *Scenedesmus* strongly increased the values of the trophic index. Species from the genera *Dinobryon*, *Kephyrion*, *Peridinium*, *Gymnodinium*, and *Oocystis* are good indicators of low trophy (Szeląg-Wasilewska et al. 1999). However, many species recorded in the lakes, such as those from the genera *Rhodomonas* and *Cryptomonas*, were found in various types of water bodies; therefore, they had no value as indicators (Hörnström 1981; Szeląg-Wasilewska et al. 1999; Reynolds et al. 2002). In lakes with the lowest trophy, the highest contribution to the phytoplankton biomass was from the centric diatoms.

In Lakes Hańcza, Szurpiły, Kamenduł, Perty, Jeglówek, Kojle, Krajwelek, and Pogorzałek, the PB values from the years 2003 (Grabowska et al. 2006) and 2009 were compared (Table 5). In a majority of cases, their values decreased. Increases in PB were only found in Lake Hańcza and Lake Pogorzałek; the increase in PB for Lake Hańcza was particularly disturbing because it is the deepest lake in Poland (Table 5).

The concentration of chlorophyll *a* in the epilimnion ranged between 1.17 μg/l (Lake Hańcza, Lake Perty, and Lake Kojle) and 8.34 μg/l (Lake Udziejek), and there was a positive correlation between the total phytoplankton biomass and chlorophyll *a* concentration (*r* = 0.871, *p* = 0.0002, *n* = 12).

An important indicator used in assessing water trophy is the primary production of phytoplankton (Wetzel 2001). The greatest value of gross primary production was observed in Lake Linówek (126.4 mg C/m^3^/h), and similar values of primary production were obtained for hypertrophic lakes with peaty-forest catchments in NE Poland (Górniak et al. 2003). The average gross production in Lake Okrągłe and Lake Hańcza was 55.5 and 31.7 mg C/m^3^/h, respectively. In Lake Hańcza and Lake Linówek, the gross primary production decreased with depth, whereas in Lake Okrągłe, an increase was found at depths of 1.5 and 4.5 m.

The community of Rotifera in the SLP lakes consisted of 38 species, and 7 to 25 species were found in particular lakes. Two species, *K. cochlearis* and *Gastropus stylifer*, occurred in all of the studies lakes (Table 6). Such a high frequency in the case of *K. cochlearis* is not surprising because it is a eurytopic species that is common in all types of waters. *G. stylifer*, however, is a species that indicates lakes with low trophic status, and it feeds exclusively on dinoflagellates by sucking out their content. A relatively high frequency (>80 %) was also noted for species of the genus *Polyarthra* (*Polyarthra remata* and *Polyarthra vulgaris*). In most of the studied lakes species characteristic for low trophy (*Asomorpha ovalis*) and high trophy (*Pompholyx sulcata*) were found as well as the large and predatory *Asplanchna priodonta*, and two species hidden in the gelatinous coating (*Collotheca mutabilis* and *Conochilus unicornis*) and eurytopic loricated *Kellicottia longispina* and *Keratella quadrata*.

**Table 6:** Lists of rotifer species in the lakes of Suwałki Landscape Park

Lakes	Species	Number
Hańcza	*Conochilus hippocrepis* (Schrank), *Filinia terminalis* (Plate), *Gastropus stylifer* Imhof, *Kellicottia longispina* (Kellicott), *Keratella cochlearis* (Gosse), *Polyarthra vulgaris* Carlin, *Synchaeta kitina* Rousselet	7
Szurpiły	*Ascomorpha ovalis* (Bergendal), *Asplanchna priodonta* Gosse, *Collotheca pelagica* (Rousselet), *Conochilus unicornis* Rousselet, *Filinia terminalis* (Plate), *Gastropus stylifer* Imhof, *Kellicottia longispina* (Kellicott), *Keratella cochlearis* (Gosse), *Keratella quadrata* (Müller), *Polyarthra major* Burckhardt, *Polyarthra remata* Skorikov, *Polyarthra vulgaris* Carlin, *Pompholyx sulcata* Hudson, *Synchaeta kitina* Rousselet, *Trichocerca capucina* (Wierzejski i Zacharias), *Trichocerca similis* (Wierzejski)	16
Kamenduł	*Ascomorpha ovalis* (Bergendal), *Ascomorpha saltans* Bartsch, *Collotheca mutabilis* (Hudson), *Conochilus unicornis* Rousselet, *Filinia longiseta* (Ehrenberg), *Gastropus hyptopus* (Ehrenberg), *Gastropus stylifer* Imhof, *Kellicottia longispina* (Kellicott), *Keratella cochlearis* (Gosse), *Keratella quadrata* (Müller), *Polyarthra major* Burckhardt, *Polyarthra remata* Skorikov, *Polyarthra vulgaris* Carlin, *Pompholyx sulcata* Hudson, *Synchaeta kitina* Rousselet, *Trichocerca similis* (Wierzejski)	16
Perty	*Ascomorpha ovalis* (Bergendal), *Asplanchna priodonta* Gosse, *Collotheca mutabilis* (Hudson), *Collotheca pelagica* (Rousselet), *Conochilus unicornis* Rousselet, *Gastropus stylifer* Imhof, *Kellicottia longispina* (Kellicott), *Keratella cochlearis* (Gosse), *Keratella quadrata* (Müller), *Polyarthra remata* Skorikov, *Polyarthra vulgaris* Carlin, *Pompholyx sulcata* Hudson, *Synchaeta kitina* Rousselet, *Trichocerca similis* (Wierzejski)	14
Jeglówek	*Ascomorpha ovalis* (Bergendal), *Ascomorpha saltans* Bartsch, *Asplanchna priodonta* Gosse, *Collotheca mutabilis* (Hudson), *Collotheca pelagica* (Rousselet), *Conochilus unicornis* Rousselet, *Gastropus stylifer* Imhof, *Kellicottia longispina* (Kellicott), *Keratella cochlearis* (Gosse), *Polyarthra remata* Skorikov, *Polyarthra vulgaris* Carlin, *Synchaeta pectinata* Ehrenberg, *Trichocerca similis* (Wierzejski)	13
Kojle	*Ascomorpha ovalis* (Bergendal), *Asplanchna priodonta* Gosse, *Collotheca mutabilis* (Hudson), *Filinia terminalis* (Plate), *Gastropus stylifer* Imhof, *Kellicottia longispina* (Kellicott), *Keratella cochlearis* (Gosse), *Polyarthra remata* Skorikov, *Polyarthra vulgaris* Carlin, *Pompholyx sulcata* Hudson	10
Okrągłe	*Anuraeopsis fissa* (Gosse), *Ascomorpha ovalis* (Bergendal), *Ascomorpha saltans* Bartsch, *Asplanchna brightwelli* Gosse, *Asplanchna priodonta* Gosse, *Collotheca mutabilis* (Hudson), *Collotheca pelagica* (Rousselet), *Conochilus unicornis* Rousselet, *Filinia longiseta* (Ehrenberg), *Gastropus stylifer* Imhof, *Kellicottia longispina* (Kellicott), *Keratella cochlearis* (Gosse), *Keratella quadrata* (Müller), *Lecane luna* (Müller), *Polyarthra major* Burckhardt, *Polyarthra remata* Skorikov, *Polyarthra vulgaris* Carlin, *Pompholyx sulcata* Hudson, *Trichocerca capucina* (Wierzejski i Zacharias), *Trichocerca pusilla* (Lauterborn), *Trichocerca rousseleti* (Voigt)	21
Krajwelek	*Anuraeopsis fissa* (Gosse), *Ascomorpha saltans* Bartsch, *Asplanchna brightwelli* Gosse, *Asplanchna priodonta* Gosse, *Brachionus quadridentatus* Hermann, *Collotheca libera* (Zacharias), *Collotheca mutabilis* (Hudson), *Collotheca pelagica* (Rousselet), *Euchlanis dilatata* Ehrenberg, *Filinia longiseta* (Ehrenberg), *Gastropus hyptopus* (Ehrenberg), *Gastropus stylifer* Imhof, *Kellicottia longispina* (Kellicott), *Keratella cochlearis* (Gosse), *Keratella quadrata* (Müller), *Lecane closterocerca* (Schmarda), *Lecane quadridentata* (Ehrenberg), *Polyarthra major* Burckhardt, *Polyarthra remata* Skorikov, *Pompholyx sulcata* Hudson, *Synchaeta kitina* Rousselet, *Synchaeta pectinata* Ehrenberg, *Trichocerca capucina* (Wierzejski i Zacharias), *Trichocerca pusilla* (Lauterborn), *Trichocerca rousseleti* (Voigt)	25
Udziejek	*Anuraeopsis fissa* (Gosse), *Asplanchna priodonta* Gosse, *Brachionus angularis* Gosse, *Collotheca libera* (Zacharias), *Collotheca mutabilis* (Hudson), *Collotheca pelagica* (Rousselet), *Conochilus unicornis* Rousselet, *Filinia longiseta* (Ehrenberg), *Gastropus hyptopus* (Ehrenberg), *Gastropus stylifer* Imhof, *Keratella cochlearis* (Gosse), *Keratella quadrata* (Müller), *Polyarthra remata* Skorikov, *Polyarthra vulgaris* Carlin, *Pompholyx complanata* Gosse, *Pompholyx sulcata* Hudson, *Trichocerca capucina* (Wierzejski i Zacharias), *Trichocerca pusilla* (Lauterborn), *Trichocerca rousseleti* (Voigt)	19
Pogorzałek	*Anuraeopsis fissa* (Gosse), *Ascomorpha saltans* Bartsch, *Asplanchna priodonta* Gosse, *Brachionus angularis* Gosse, *Collotheca mutabilis* (Hudson), *Conochilioides dossuarius* (Hudson), *Conochilus unicornis* Rousselet, *Filinia longiseta* (Ehrenberg), *Filinia terminalis* (Plate), *Gastropus hyptopus* (Ehrenberg), *Gastropus stylifer* Imhof, *Kellicottia longispina* (Kellicott), *Keratella cochlearis* (Gosse), *Keratella hiemalis* Carlin, *Keratella quadrata* (Müller), *Polyarthra dolichoptera* Idelson, *Polyarthra remata* Skorikov, *Polyarthra vulgaris* Carlin, *Pompholyx sulcata* Hudson, *Trichocerca capucina* (Wierzejski i Zacharias)	20
Linówek	*Ascomorpha ovalis* (Bergendal), *Asplanchna priodonta* Gosse, *Collotheca mutabilis* (Hudson), *Filinia longiseta* (Ehrenberg), *Gastropus stylifer* Imhof, *Keratella cochlearis* (Gosse), *Polyarthra remata* Skorikov, *Polyarthra vulgaris* Carlin, *Trichocerca capucina* (Wierzejski i Zacharias), *Trichocerca similis* (Wierzejski)	10

Four species had the highest frequency in the epilimnion of the studied lakes and consisted of approximately 78 % of the whole rotifer community; their contribution to the rotifer numbers ranged from 53 to 93 %. In the remaining two layers, differences were higher among three lakes: in the metalimnion of Lake Hańcza, 84 % of the rotifer numbers were from *Conochilus hippocrepis*; in Lake Udziejek, two species that were absent in the epilimnion dominated in the near-bottom layer (*Gastropus hyptopus* (21 %) and *Pompholyx complanata* (24 %)); and in Lake Pogorzałek, this layer was dominated by *Filinia longiseta* (68 %).

A characteristic feature of all of the studied lakes is the presence of at least one species from the list of species indicative of low trophy (Table 7). In nearly all of the lakes, *G. stylifer* occurred, and it was accompanied by *A. ovalis* in six lakes. Three remaining species were less frequent, and *C. hippocrepis* was recorded in only one lake (Lake Hańcza). Species that indicate high trophy did not occur at all in Lake Jeglówek and Lake Hańcza, whereas in Lake Udziejek, six of the seven species of this group were found.

**Table 7:** Share (percent) of low and high trophy indicatory species in the total numbers of Rotifera

	Hańcza	Szurpiły	Kamenduł	Perty	Jeglówek	Kojle	Okrągłe	Krajwelek	Udziejek	Pogorzałek	Linówek
Indicatory species of low trophic status											
* Ascomorpha ovalis*		2.6	2.8	1.8	0.7	2.5	0.2				0.7
* Conochilus hippocrepis*	28.2										
* Gastropus stylifer*	0.4	7.0	6.9	2.4	14.2	12.7		0.6	0.6	1.6	12.5
* Polyarthra major*		2.6					0.2				
Total	28.6	11.6	9.7	4.2	14.9	15.2	0.4	0.6	0.6	1.6	13.2
Indicatory species of high trophic status											
* Anuraeopsis fissa*										1.6	
* Brachionus angularis*									0.8		
* K. cochlearis tecta*		1.7	15.3				21.9	30.3	12.0	1.6	
* Filinia longiseta*			25.0				3.0	1.7	2.0		1.9
* Keratella quadrata*							0.2		3.6		
* Pompholyx sulcata*				4.7		2.5	2.2		1.5	17.4	
* Trichocerca pusilla*							0.6		1.0		
Total	0	1.7	40.3	4.7	0	2.5	27.9	32.0	20.9	20.6	1.9

**Fig. 8:**
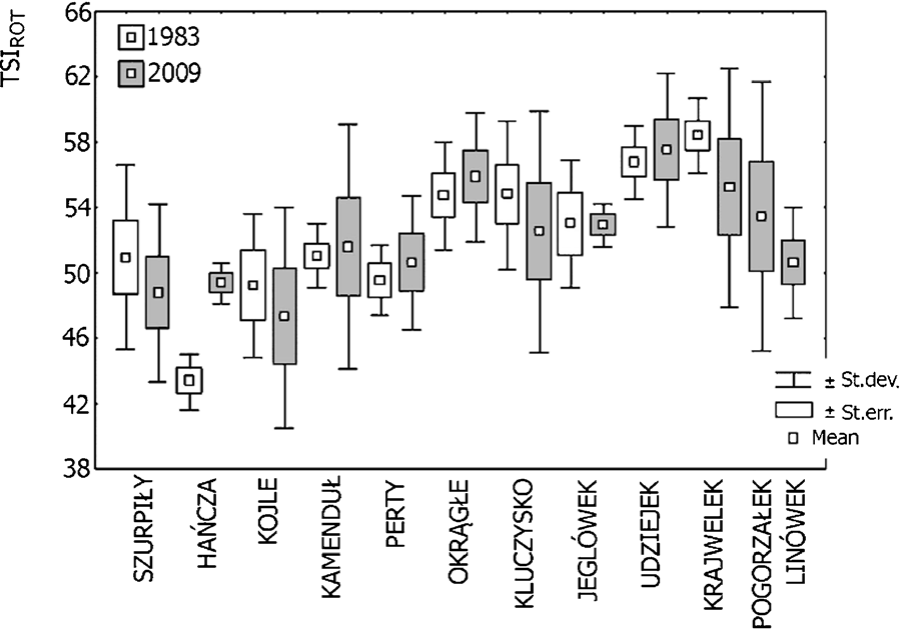
Values of TSI_ROT_ in the selected lakes of Suwałki Landscape Park

Considering all of the indices of trophic state based on the analysis of the structure of rotifer community (Ejsmont-Karabin 2012), the studied group of lakes consisted of mesoeutrophic and eutrophic water bodies (Fig. 8). The only mesotrophic lake in 1983 was Lake Hańcza, and it had a higher trophic status in 2009, which indicated its mesoeutrophy. Among the lakes that underwent eutrophication over the 26 years of research, there were lakes that retained the same trophic status (i.e., mesoeutrophic Lakes Kamenduł and Perty and eutrophic Lake Udziejek) and one that shifted from mesoeutrophy to eutrophy (Lake Okrągłe).

Another group of lakes was characterized by a decrease in trophic status and included mesoeutrophic Lakes Szurpiły and Kojle and eutrophic Lake Krajwelek (Fig. 8). In Lake Jeglówek, no significant changes in trophy were observed. In the two remaining lakes, research was only carried out in 2009.

The species composition and biomass of phytoplankton and zooplankton as well as the population characteristics of single species represent important inputs when studying the effects of man-made environmental changes in lake ecosystems (Schindler 1987). Zooplankton represents an important trophic level and is detrimental to the flow of matter and energy (Sommer et al. 1986). Zooplankton indices based on taxonomic composition, size distribution, trophic levels, spatial patterns, functional characteristics, and quantitative data may be of high informative potential and should not be overlooked in monitoring studies (Andronikova 1996).

In the pelagic zone of the studied lakes, 36 species of Crustacea were found, with Cladocera represented by 24 taxa and Copepoda by 12 taxa. Particularly noteworthy is the species abundance of genus *Bosmina* (seven species) and presence of rare planktonic species such as *Heterocope appendiculata* (Lake Szurpiły and Lake Kameduł), *Bythotrephes longimanus* (Lake Hańcza) and *Daphnia hyalina* (Lake Perty). However, a few dominant species (*Thermocyclops oithonoides*, *Mesocyclops leuckarti*, *Daphnia cucullata*, *Bosmina longirostris*, *Diaphanosoma brachyurum*, and *Eudiaptomus graciloides*) were the most numerous. The dominant species position shifting, quantitative data, and functional characteristics are good indices of the eutrophication process. The density, biomass, and taxonomic structure of pelagic Crustacea were different in the epilimnion of the studied lakes. According to Karabin’s crustacean zooplankton indices TSI_CRU_ (Table 8), the following trophic levels were distinguished: mesotrophic lakes (Lake Hańcza and Lake Szurpiły), mesoeutrophic lakes (Lake Okrągłe, Lake Pogorzałek, Lake Perty, Lake Kojle, Lake Jeglówek, and Lake Kamenduł), and eutrophic lakes (Lake Krejwelek, Lake Udziejek, and Lake Linówek). Tables 8 and 9 present the selected zooplankton community indices proposed in the literature for monitoring eutrophication processes. Lakes with lower trophic status have less numerous crustacean zooplankton with a greater diversity of species. However, in these lakes, the average biomass was the highest (Table 9) because of the presence of larger zooplankton species. Therefore, the individual mean biomass for the entire zooplankton community (*W*) appears to be a good index of eutrophication. Mesotrophic and mesoeutrophic lakes have a higher biomass of zooplankton than phytoplankton, and this factor combined with the domination of herbivorous crustacean zooplankton (Table 9) indicates good conditions in these lakes. Eutrophic lakes have a larger share of organisms that feed on smaller particles such as the suspension of bacteria and detritus.

**Table 8:** Crustacea trophic state index (TSI_CRU_) according to Karabin (1985) and selected characteristics of the crustacean zooplankton structure in the lakes of Suwałki Landscape Park

Lakes	TSI_CCB_	TSI_BR_	TSI_IS_	TSI_CRU_	*A*	*N*	*W* = *B* / *N*	*B*_Cycl_/*B*_Cal_	*N*_Cla_/*N*_Cop_	*B*_zoo_/*B*_phyto_
Hańcza	45.5	44.0	40.6	43.4	73	62	0.051	7.50	1.0	2.7
Szurpiły	38.4	38.9	40.9	39.4	89	35	0.041	0.74	1.5	1.1
Kamenduł	55.5	58.2	44.4	52.7	72	22	0.023	1.57	0.13	0.2
Perty	42.7	51.3	51.0	48.3	77	124	0.023	0.22	0.33	2.46
Jeglówek	49.1	59.5	47.7	52.1	64,6	41	0.015	1.31	2.16	0.49
Kojle	46.8	49.1	52.4	49.4	53	55	0.034	0.50	0.46	1.6
Okrągłe	46.0	45.3	45.1	45.5	67	117	0.029	1.84	0.69	0.6
Krajwelek	66.4	64.2	60.1	63.6	0	20	0.016	41.00	0.51	0.07
Udziejek	59.4	58.3	47.5	55.1	61	157	0.013	0	0.26	0.25
Pogorzałek	47.0	45.5	50.8	47.8	58	59	0.018	6,6	0.61	–
Linówek	57.0	59.0	57.1	57.5	35	101	0.021	2.16	0.16	0.85

*A* percentage of herbivorous in total Crustacea biomass (percent), *N* number of Crustacea (individuals per liter), *B* biomass of Crustacea (milligrams per liter), *W* individual mean biomass in the whole zooplankton community (milligrams), *B*_*Cycl*_ */B*_*Cal*_ biomass of Cyclopoida to biomass of Calanoida, *N*_*Cla*_ */N*_*Cop*_ numbers of Cladocera to numbers of Copepoda, *B*_*zoo*_ */B*_*phyto*_ biomass of crustacean zooplankton to biomass of phytoplankton

**Table 9:** Mean value for the selected parameters of crustacean zooplankton community in the lakes of different trophic statuses

Characteristics of crustacean zooplankton communities	Mesotrophic lakes	Mesoeutrophic lakes	Eutrophic lakes
Percentage of biomass herbivorous Crustacea	81	65	32
Numbers (*N*) [individuals/l]	49	70	93
Biomass (*B*) [mg/l]	2.33	1.75	1.56
Individual mean biomass in the whole zooplankton community, *W* = *B* / *N* [mg]	0.046	0.024	0.017
*N*_Cladocera_/*N*_Copepoda_	1.25	0.73	0.31
*B*_zooplankton_/*B*_phytoplankton_	1.9	1.07	0.39
Number of species	14.5	10.5	8

The share of Cyclopoida in the structure of crustacean zooplankton is one of the main indicators of the trophic state of lakes proposed by Karabin (1985) and Ejsmont-Karabin and Karabin (2013). In the mesotrophic lakes, the Cyclopoida share of the total biomass of crustacean zooplankton did not exceed 15 %, and Cladocera was the dominant group. The share of the order Cyclopoida increases in more productive lakes (*N*_Cla_/*N*_Cop_, Table 8). More productive lakes usually have a lower share of Calanoida in the subclass Copepoda (*B*_Cycl_/*B*_Cal_, Table 8). Copepods, especially the calanoids, are the dominant crustaceans in the communities of the pelagic zone of large lakes under mesooligotrophic conditions (Sager and Richman 1991; Carter et al. 1995; Andronikova 1996; Rahkola-Sorsa 2008).

Zooplankton is a community that consists mostly of herbivores and bacterivores and may represent a sensitive indicator of the trophic status of lakes. The values obtained by the zooplankton indices showed similar trophic states as the Carlson indices (TSI) in the studied lakes. Parameters of zooplankton may indicate the processes occurring in the lake; however, these parameters are difficult to detect by chemical assay alone (Karabin 1985).

## Summary

The SLP lakes are of postglacial origin, and the deepest (Lake Hańcza) represents a channel type. From a hydrological perspective, most of the investigated lakes are classified as flowing and all of them belong to the Czarna Hańcza and Szeszupa catchment area. The water quality of lakes depends on the supply intensity (surface and groundwater), rate of water exchange, and biological and chemical processes in lacustrine ecosystems. The geological structure of the area and land development shaped the trophic status of the studied lakes.

Over the past 30–40 years, no major changes in the hydrochemistry of the lakes of Suwalki Landscape Park were recorded. Compared with neighboring lakes, the SLP lakes are characterized by good oxygenation of the surface layer and higher concentrations of calcium and magnesium ions, which affect the value of proper conductivity. The concentrations of biogenic elements in the water of the SLP lakes are much lower than in other lakes of the region. Maintaining a low trophic level in the studied lakes is possible because of the large buffering properties and low phosphorus loads from the catchment. Despite increasing pressure from tourism and recreation in the environment, signs of increased eutrophication are not observed. This is shown by the concentrations of biogenic elements (total phosphorus) and TSI_TP_, which had slightly lower values than at the beginning of the twenty-first century. A comparison of the calculated trophic status indices for much smaller lakes based on biological indicators confirms the high quality of this environment (Table 10).

**Table 10:** Trophic status in the lakes of Suwałki Landscape Park according to different indices

Lakes	TSI	BN	TI_PB_	TSI_ROT_	TSI_CRU_
Hańcza	M	E	M	M/E	M
Szurpiły	E	M	M	M/E	M
Kamenduł	E	E	M/E	M/E	M/E
Perty	M	E	M	M/E	M/E
Jeglówek	M	E	M	M/E	M/E
Kojle	E	E	M	M/E	M/E
Przechodnie	E	E	M/E	–	–
Okrągłe	E	E	M	E	M/E
Krajwelek	E	E	M/E	E	E
Udziejek	E	E	E	E	E
Pogorzałek	E	E	M/E	M/E	E
Linówek	E	H	M/E	M/E	E

*M* mesotrophic lake, *E* eutrophic lake, *H* hypertrophic lake

The trophic status of the investigated SLP lakes was determined according to the bacterial numbers and showed a lack of oligotrophic lakes in the area. Only Lake Szurpiły can be classified as mesotrophic, and Lake Linówek can be characterized as hypertrophic with some features typical for humic water.

Five stratified lakes that were dominated or codominated by diatoms, chlorophytes, and chrysophytes represented the lowest trophic level, i.e., mesotrophic. Other lakes with a higher share of Cyanoprokaryota were classified as mesoeutrophic (5) and eutrophic (1).

The parameters of zooplankton showed a similar trophic status as the Carlson indicators (TSI) in the studied lakes and are capable of indicating the processes that may occur in the lake; however, they are difficult to detect by chemical assay alone. Zooplankton is an important level of the trophic chain in freshwater ecosystems and a good indicator of changes in lakes; however, it was not considered a useful index in the Water Framework Directive.

In conclusion, several metrics were used to describe trophic state of lakes, but only a few fulfilled the requirement of being a good indicator of eutrophication.
